# Tissue engineering of acellular vascular grafts capable of somatic growth in young lambs

**DOI:** 10.1038/ncomms12951

**Published:** 2016-09-27

**Authors:** Zeeshan Syedain, Jay Reimer, Matthew Lahti, James Berry, Sandra Johnson, Richard Bianco, Robert T. Tranquillo

**Affiliations:** 1 Department of Biomedical Engineering, University of Minnesota, Minneapolis, Minnesota 55455, USA; 2 Experimental Surgical Services, Department of Surgery, University of Minnesota, Minneapolis, Minnesota 55455, USA; 3 Department of Chemical Engineering & Materials Science, University of Minnesota, Minneapolis, Minnesota 55455, USA

## Abstract

Treatment of congenital heart defects in children requiring right ventricular outflow tract reconstruction typically involves multiple open-heart surgeries because all existing graft materials have no growth potential. Here we present an ‘off-the-shelf' vascular graft grown from donor fibroblasts in a fibrin gel to address this critical unmet need. In a proof-of-concept study, the decellularized grafts are implanted as a pulmonary artery replacement in three young lambs and evaluated to adulthood. Longitudinal ultrasounds document dimensional growth of the grafts. The lambs show normal growth, increasing body weight by 366% and graft diameter and volume by 56% and 216%, respectively. Explanted grafts display physiological strength and stiffness, complete lumen endothelialization and extensive population by mature smooth muscle cells. The grafts also show substantial elastin deposition and a 465% increase in collagen content, without signs of calcification, aneurysm or stenosis. Collectively, our data support somatic growth of this completely biological graft.

## Abstract (web summary)

Current vessel grafts must be surgically replaced when the recipient outgrows them. Here, Syedain *et al.* bioengineer a tube of acellular matrix produced from sheep fibroblasts that is capable of cellularizaton and somatic growth when transplanted into growing lambs, eliminating the need for multiple graft surgeries.

## Graphical abstract

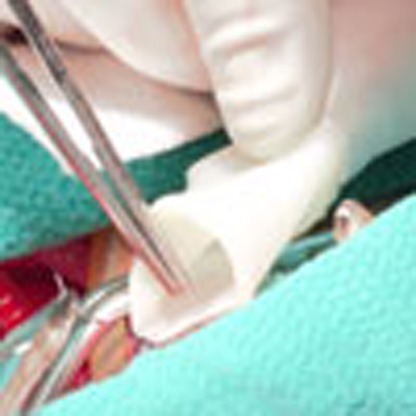

The incidence of surgical correction of congenital heart defects has increased dramatically over the last several decades. These defects, considered fatal just 30 years ago, can often be corrected successfully with overall operative mortality of <2% (refs 1, 2). Reconstruction or replacement of blood vessels, valves and cardiac chambers is frequently required to repair or reform the appropriate anatomic configuration. The use of synthetic materials, with zero growth potential and unpredictable durability, is often the only way to achieve these operative goals. The availability of tissue-engineered material, with the ability to grow, heal and provide long-term durability, would revolutionize the practice of congenital heart surgery.

Tetralogy of Fallot and pulmonary atresia with ventricular septal defect are just two examples of cardiac defects that, despite excellent long-term survival typically require multiple operative procedures to replace the reconstructed connection between the right ventricle and pulmonary artery. Currently, homograft pulmonary artery conduits or bovine jugular vein grafts are the only materials able to create this connection. These conduits have no ability to grow and remodel with the somatic growth of the child. Additionally, an intense inflammatory reaction to these materials commonly occurs, resulting in early calcification and failure^3^. Thus, these patients will sometimes require five to seven operative procedures during their lifetimes even with a ‘successful' corrective procedure^4^. Although a valved conduit would benefit a larger patient population, and several research groups are working on such grafts^5^,^6^,^7^,^8^,^9^,^10^, a conduit with the ability to grow somatically would suffice for many patients with competent valves who require cardiopulmonary vascular reconstruction. It might also be a compromise solution for patients in need of a valved conduit absent the availability of one with growth potential.

The application of a competent, readily available conduit, with the ability to grow with the child, would eliminate the need for multiple operations and the morbidities associated with these procedures, possibly benefiting more than 1,000 pediatric patients annually in the USA^4^. It would also dramatically reduce the financial burden on the health care system associated with currently used conduits that require periodic replacement to accommodate child growth.

Pioneering research in this field has been conducted by Shin'oka *et al.*^11^ with the landmark report of reconstruction of an occluded pulmonary artery in a 4-year old patient with a degradable synthetic polymer tube of the copolymer polylactic acid/polycaprolactone seeded with autologous cells. In a subsequent clinical trial, similar grafts seeded with autologous bone marrow mononuclear cells were implanted into 42 patients (median age 5.5 years)^12^. There was no graft-related mortality with mean follow-up of 5.8 years although one patient had a partial mural thrombosis, and four patients had graft stenosis^13^. Histological examination of one graft was conducted after 12 years in a patient (implanted at age 4 years), showing graft remodelling with complete lumen endothelialization and a mature smooth muscle wall^14^. In addition, investigators have conducted extensive research in the mouse aorta model to elucidate the role of the seeded cells and the host response^15^,^16^,^17^,^18^,^19^,^20^,^21^,^22^.

In a sheep study using autologous cells seeded on a synthetic polymer scaffold, Hoerstrup *et al.*^23^,^24^ implanted 18 mm diameter polylactic acid/poly-4-hydroxybutarate tubes seeded with autologous myofibroblasts into lambs as arterial replacements for up to 240 weeks. Longitudinal computed tomography imaging and explant histology revealed extensive remodelling and graft growth.

Both of the above approaches, although successful in preclinical (Hoerstrup *et al.*) and clinical (Shin'oka *et al.)* studies, require autologous cells to be isolated from the patient and expanded before implantation. An ‘off-the-shelf' graft that is acellular and possesses growth potential via host cell invasion post-implantation, as reported herein, would be a substantial clinical advance. In addition, an acellular graft would eliminate the need to develop a manufacturing process to isolate autologous cells and reliably seed them onto the scaffold in order to translate the technology into the clinic.

These completely-biological tubular grafts, consisting primarily of cell-produced collagen, are derived from a sacrificial fibrin gel, which is remodelled in a bioreactor by entrapped dermal fibroblasts. After remodelling, the tubes are decellularized, which, when done effectively, removes the need for immunosuppression, but maintains the cell-produced matrix. The graft is grown to be strongly aligned in the circumferential direction in order to mimic the mechanical anisotropy associated with native arteries.

The goal of this study is to demonstrate proof-of-principle for an ‘off-the-shelf' graft that is capable of somatic growth and can thus serve as a conduit for pediatric cardiac surgeries. These acellular allografts are characterized, stored, and then implanted into three lambs (average age 8 weeks), tracked longitudinally with ultrasound, and then explanted after the lambs reach adult size (age 50 weeks) for mechanical, biochemical and histological characterization. These characterizations are performed in order to answer key questions about the graft including immunogenicity, propensity for calcification, matrix remodelling in relation to maintenance of stiffness and strength, capacity for recellularization and somatic growth potential.

## Results

### Tissue-engineered arterial graft

**Figure 1:**
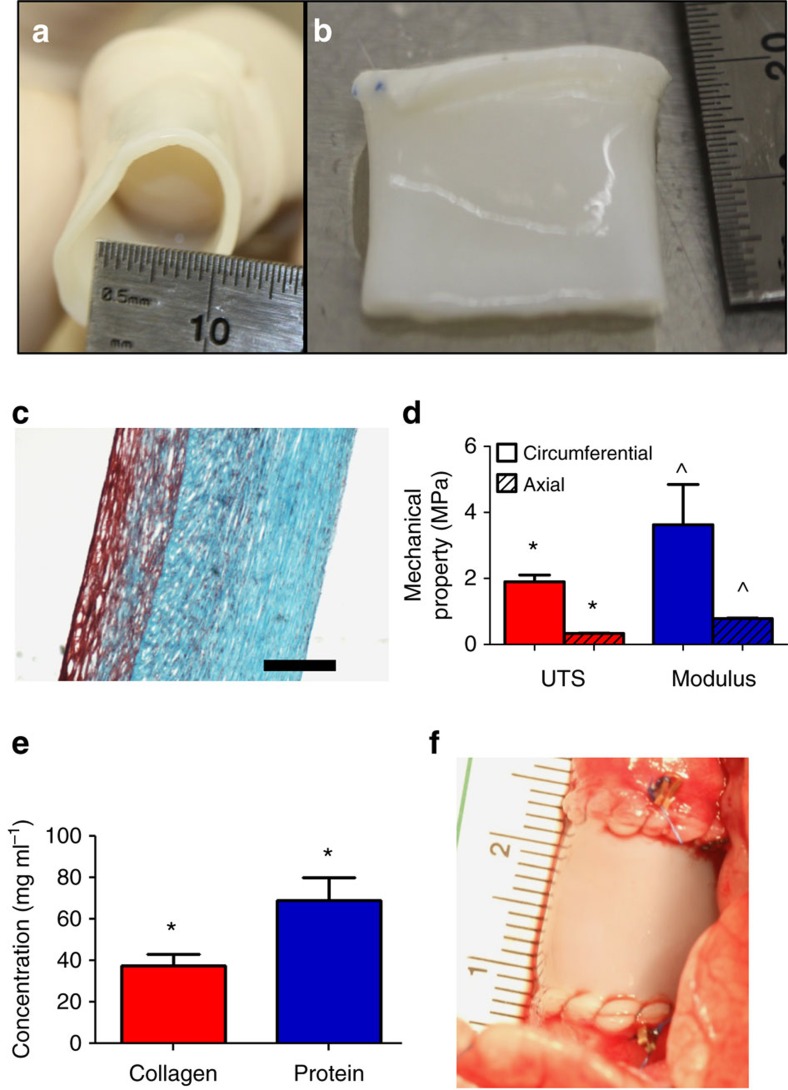
Images of decellularized tissue-engineered graft. (**a**) End-on view and (**b**) side view. (**c**) Trichrome stained circumferential section image of the graft (scale bar, 200 μm in black). (**d**) Tensile mechanical properties of the graft in the circumferential (solid) and axial (dashed) directions (*n*=5). (**e**) Collagen and total protein concentrations of the graft before implantation (*n*=5). (**f**) Image of graft implanted in the ovine pulmonary artery with biodegradable sutures and silver clip markers near the anastomoses. s.d. is reported as error bars, with paired symbols indicate differences at *P*<0.05 using Student *t*-test.

Decellularized tissue-engineered tubes (16 mm inner diameter) were evaluated for tensile mechanical, suture retention and biochemical properties. The resulting tubular grafts had thicknesses of 1.21±0.03 mm, which was comparable to the pulmonary artery (1.15±0.20 mm). A representative end and side view of the graft are shown Fig. 1a,b. Histologically, the grafts were predominantly collagen with a layer of residual fibrin on the lumenal surface (Fig. 1c). Stretch-to-failure testing showed the grafts possessed an ultimate tensile strength (UTS) of 1.9±0.2 MPa and stiffness of 3.6±1.2 MPa (Fig. 1d) in the circumferential direction. The tissue tubes also possessed mechanical anisotropy, with stiffness being 4.5-fold higher in the circumferential direction than the axial direction. The suture retention strength with 7-0 prolene suture was 175±56 g. All of these values compared favourably to the ovine pulmonary artery, including suture retention strength of 224±31 g for native pulmonary artery. The total collagen concentration before implantation was 38±5 mg ml^−1^ (Fig. 1e). The DNA content after decellularization was 1±1% of the content before decellularization (from 422±59 ug ml^−1^ of tissue volume to 5.5±7.8 ug ml^−1^).

The grafts were implanted interpositionally into the pulmonary artery (Fig. 1f), following resection of a similar length of native artery, in three lambs. 5-0 Maxon CV biodegradable sutures (*in vivo* strength half-life of 4 weeks) were used to attach the graft to the pulmonary artery at the anastomoses. In addition, to ensure anastomoses could be identified at explant, silver clips were sewn on top of the native artery near each anastomosis (Fig. 1f).

### Growth evaluation of pulmonary artery graft with ultrasound

**Figure 2:**
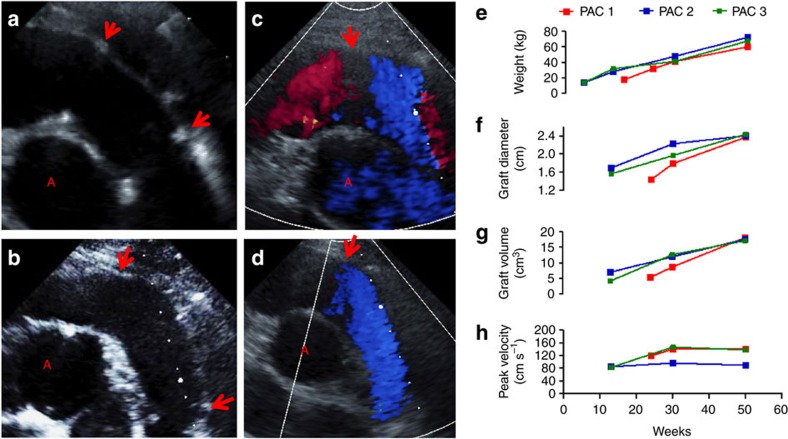
Representative ultrasound images of the grafts post-implantation. (**a**) After 8 weeks implantation and (**b**) at animal age of 50 weeks, including colour Doppler flow in the graft (**c**) at proximal anastomosis and (**d**) through the entire graft, with red arrows pointing to the anastomoses and the ‘A' indicating the cross-section of the aorta. (**e**) Animal weights and ultrasound measurements for (**f**) mid-graft diameter, (**g**) graft volume and (**h**) peak blood velocity in the graft as a function of animal age.

All animals were first evaluated with ultrasound at 8 weeks following implantation to assess graft diameter, graft length, blood velocity in the graft and right heart function. Figure 2a shows a representative image of the graft 8 weeks after implant with red arrows indicating the anastomoses. The grafts, although originally implanted as straight tubes, showed curvature similar to the native pulmonary artery after 8 weeks. The second and third ultrasounds were performed when the lambs reached the age of 30 and 50 weeks, respectively. Figure 2b shows a representative image of the grafts at 50 weeks, with prominent curvature, no indication of calcification (speckles in ultrasound), and no evidence of graft stenosis or aneurysm. At 50 weeks, there was no difference in diameter of the graft and adjacent native artery (Fig. 2c) leading to laminar flow through the graft (Fig. 2d). This was true for all three grafts (Supplementary Fig. 1). All animals had healthy weight gains with an increase of 340% over the course of study (Fig. 2e). The mid-graft diameter increased over the course of implantation by 56% (Fig. 2f) and total volume of the graft increased by 216%, as determined by measurements in ultrasound (Fig. 2g). There was no pathological increase in flow velocity measured within the graft during the study duration, indicating a lack of stenosis (Fig. 2h). Further, there was no measured increase/decrease in velocity downstream of the graft indicating no pressure gradient across the graft. The right heart function was also normal with no change in wall motion or pulmonary valve function.

### Explanted graft gross pathology

**Figure 3:**
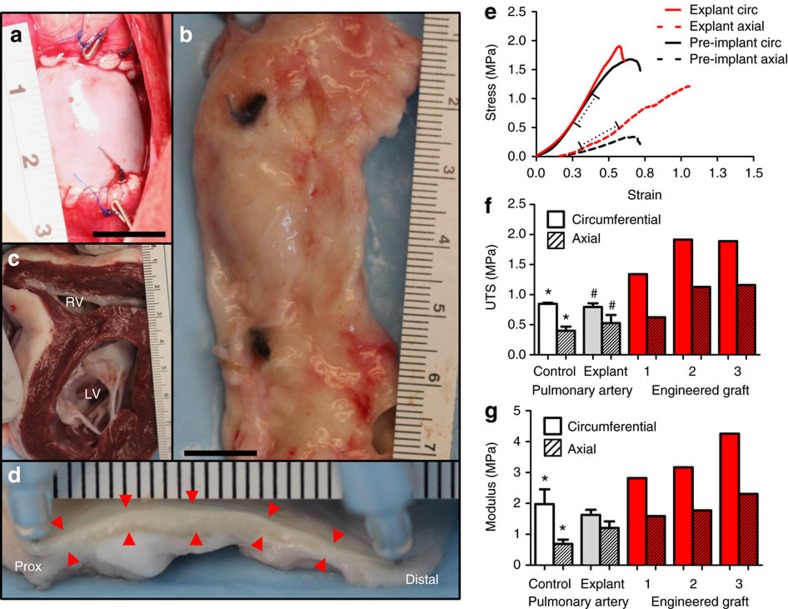
Macroscopic images of the graft at the same magnification suggesting anatomical growth. (**a**) Immediately after implantation, and (**b**) after explantation. (**c**) Cross-section of the explanted heart showing normal right ventricular wall thickness. (**d**) Side view of the explanted graft showing uniform thickness (red arrows point to the lumenal and ablumenal surfaces). (**e**) Stress–strain plots of representative pre-implant (black line) and explanted grafts in the circumferential (solid) and axial (hashed) directions, dashed segment showing linear region where modulus was calculated, and (**f**) UTS and (**g**) modulus for the three explanted grafts and the adjacent pulmonary artery (‘Explant') (*n*=3) along with non-surgical age-matched control (*n*=3) pulmonary artery (‘Control') in the circumferential (solid) and axial (dashed) directions. s.d. is reported as error bars, with paired symbols indicate differences at *P*<0.05 using Student *t*-test. Since all explanted grafts were plotted individually, no statistical comparison with them was performed.

All grafts were explanted when the animals were anatomically mature at age of 50 weeks. Grossly, the explanted grafts looked larger in both diameter and length when compared with the pre-implant graft. Figure 3a,b shows the pre-implanted and explanted grafts at the same scale (Supplementary Fig. 2 shows images of all grafts at implant and explant). The measured diameter and length of each graft at implant and explant is reported in Table 1, and the diameter compares well with that of the pulmonary artery in age-matched 1-year-old non-surgical sheep measured to be 22.3±2.1 mm (*n*=3). The explanted grafts had a diameter consistent with the adjacent pulmonary artery (Fig. 3b). The average wall thickness of the right ventricle was 6.3±0.6 mm (Fig. 3c) and that in non-surgical sheep was 7.2±0.3 mm (c.f. Supplementary Fig. 2g,h). Cross-sections of the explanted graft showed homogeneous thickness across the length (Fig. 3d, red arrows indicating lumenal and ablumenal surfaces). The anastomotic regions had no scarring or stenotic tissue and the lumenal surface transitioned smoothly from the native artery to the engineered graft.

### Explanted graft mechanical and biochemical properties

**Figure 4:**
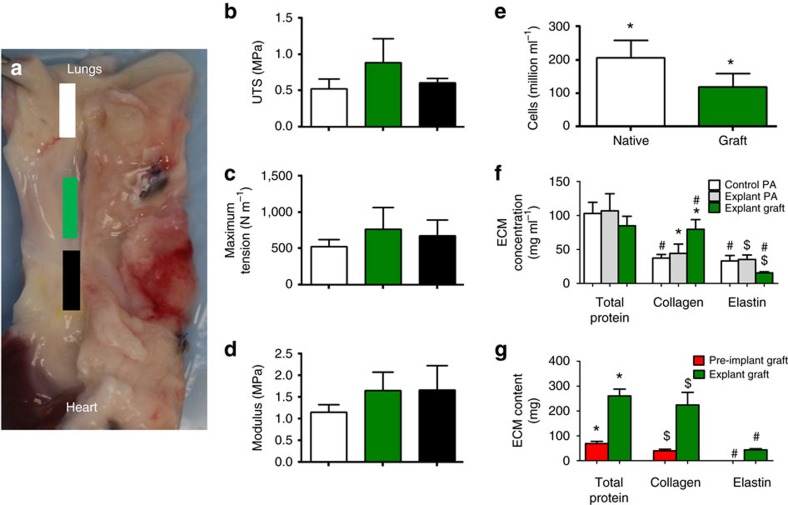
Mechanical properties and composition of the grafts indicating anatomical growth. Tensile properties in the axial direction of explant strips tested from (**a**) adjacent pulmonary artery (white), region encompassing the anastomosis (green) and engineered graft (black). Measured properties from all three grafts are averaged and s.d. reported as error bars for (**b**) UTS, (**c**) maximum tension and (**d**) modulus, with no statistical differences at *P*<0.05 using analysis of variance with Tukey *post-hoc* analysis. Comparison between the non-surgical age-matched pulmonary artery, explanted pulmonary artery and the explanted grafts (*n*=3 for all groups) of (**e**) cellularity and (**f**) extracellular matrix (ECM) protein concentrations. (**g**) Total protein, collagen and elastin content in the engineered grafts before and after implantation. s.d. is reported as error bars. Paired symbols show differences between groups at *P*<0.05 using Student *t*-test.

The explanted grafts were cut into strips and stretched to failure. Graft thickness was 0.85±0.09 mm as compared with explanted pulmonary artery thickness of 1.15±0.2 mm and control pulmonary artery thickness of 1.26±0.14 mm. The explanted graft stress–strain curves in the circumferential and axial directions, along with the pre-implant graft curves, are shown in Fig. 3e. The graft UTS and modulus in the circumferential direction were 1.5–2 fold higher than the age-matched control and explanted pulmonary artery (Fig. 3f,g) with UTS in the circumferential direction of 1.6±0.5 MPa and modulus of 3.3±0.8 MPa. The modulus was 1.8-fold higher in the circumferential direction compared with the axial direction. In order to assess how mechanically robust the fusion at the anastomoses was and any influence of scar tissue formation, axial strips from this region were compared with axial strips from the graft and the adjacent pulmonary artery (Fig. 4a). UTS, maximum tension and modulus were not different between the three regions (Fig. 4b–d).

The explanted graft DNA content was 58% of the pulmonary artery value, corresponding to a cell concentration of 119±15 million cells ml^−1^ (Fig. 4e). The total collagen and elastin content in the grafts were 180% and 49% of the pulmonary artery, respectively (Fig. 4f). The total protein content was 80% of the pulmonary artery (Fig. 4f). Based on collagen concentration, total surface area, and thickness measured for implanted and explanted grafts, the total collagen content of the explanted grafts was 224±51 mg, which was 465% higher than total collagen content of the pre-implanted grafts (40±7 mg). There was also 277% increase in total protein content of tissue. Compared with pre-implant grafts, which had no detectable level of elastin, explanted grafts contained substantial elastin (Fig. 4g). As for the tensile mechanical properties, there were no differences in compositional measures between the control and explanted pulmonary artery groups.

### Explanted graft histological analysis

**Figure 5:**
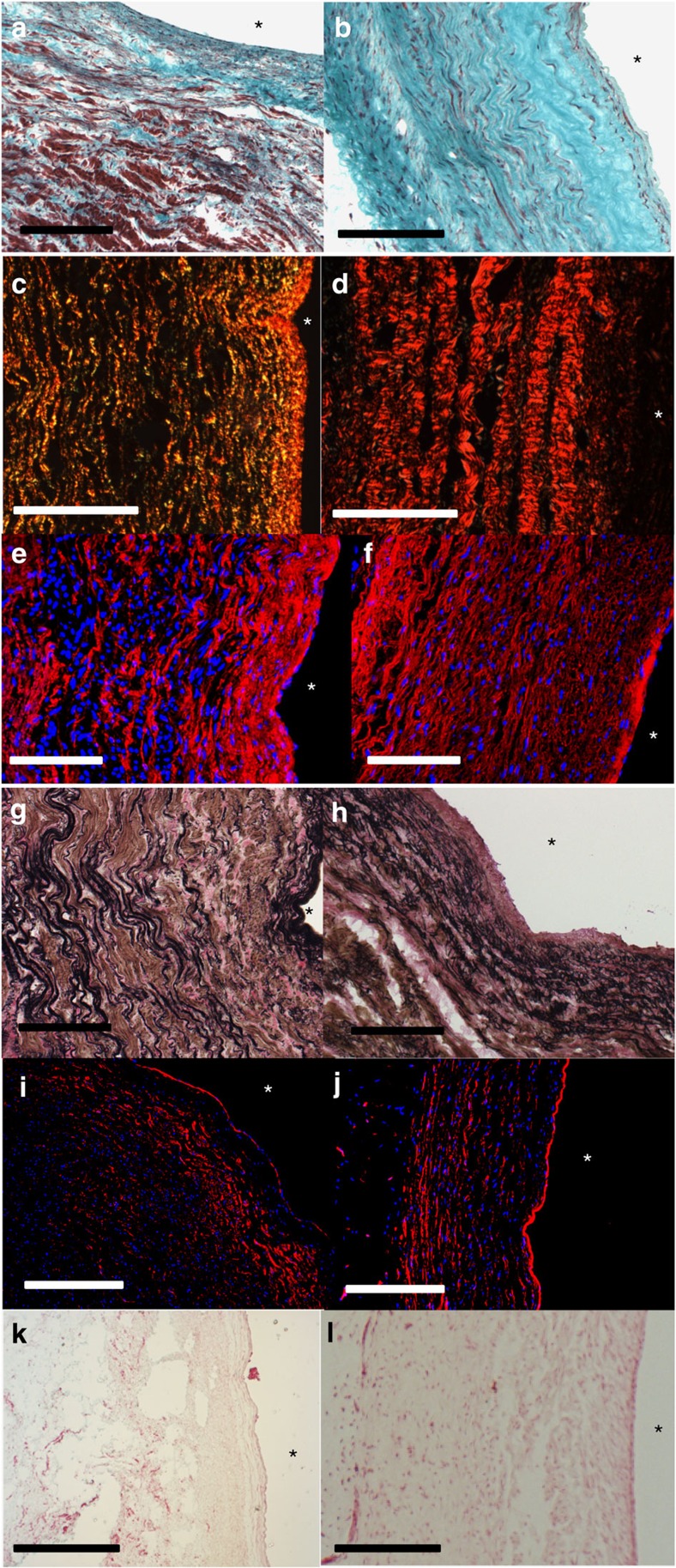
Histological images of the engineered graft and adjacent pulmonary artery following explant. Trichrome images of the (**a**) artery and the (**b**) graft showing uniform cell density and abundant collagen staining. Picrosirius red staining imaged under polarized light shows collagen crimping both in the (**c**) artery and (**d**) graft. Elastin immunostaining for (**e**) artery and (**f**) graft show distribution of elastic fiber in the entire thickness of the graft, and Verhoeff stain for elastin showing mature fibers near the lumenal surface in black both for (**g**) artery and (**h**) graft. Collagen IV immunostaining at the lumenal surface both for the (**i**) artery and (**j**) graft. No calcification was observed in the (**k**) artery and (**l**) graft as visualized by Von Kossa staining. (**a**–**l**) are circumferential sections. Scale bar, 200 μm in black or white is shown. Lumenal surface is marked with ‘*'.

The explanted graft and pulmonary artery sections were stained to visualize the matrix composition. Trichrome staining showed the multi-layer nature of the pulmonary artery (Fig. 5a) and complete remodelling of the lumenal fibrin layer into a dense collagen network and evidence of recellularization along the entire graft's length (Fig. 5b). Further characterization with picrosirius red staining imaged under polarized light showed crimped collagen fibers similar to the pulmonary artery (Fig. 5c,d). Elastin was present throughout the entire pulmonary artery (Fig. 5e) and graft (Fig. 5f), with evidence of mature elastin in the pulmonary artery (Fig. 5g) and near the lumenal surface of the graft (Fig. 5h). The basement membrane protein collagen IV was strongly expressed at the lumenal surface of the pulmonary artery (Fig. 5i) and the graft (Fig. 5j). Von Kossa staining showed no evidence of calcification along the entire length of the grafts and adjacent native artery (Fig. 5k,l), except at localized sites near the anastomoses in the adjacent pulmonary artery where the sutures had degraded.

**Figure 6:**
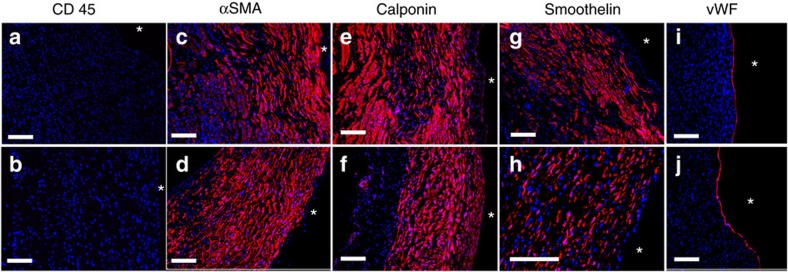
Immunostaining for cell markers in engineered graft and adjacent pulmonary artery following explant. (**a**,**c**,**e**,**g**,**i**) Explanted pulmonary artery. (**b**,**d**,**f**,**h**,**j**) Explanted engineered graft. Specific cell markers stained include (**a**,**b**). CD45, (**c**,**d**) α-SMA, (**e**,**f**) calponin, (**g**,**h**) smoothelin and (**i**,**j**) von Willebrand factor (vWF). Scale bar, 200 μm in white is shown. (**a**–**h**) are circumferential sections, **i**,**j** are axial sections. Lumenal surface is marked with ‘*'.

Immunostaining was performed in order identify the phenotype of the invaded host cells and then counterstained with Hoechst. Recellularization across the entire thickness and length of the grafts was observed (Fig. 6). The absence of CD45-expressing cells indicated a lack of immune cell types in the pulmonary artery (Fig. 6a) and the explanted graft (Fig. 6b).

Many cells present in the graft stained positive for α-smooth muscle actin (α-SMA), calponin, and smoothelin (Fig. 6d,f,h), which are markers for mature smooth muscle cells, with smoothelin reported to be definitive in this respect^25^,^26^. Similar staining was also seen in the native pulmonary artery (Fig. 6c,e,g). In both the graft and native artery, few cells stained positive for the proliferative marker Ki67, indicating a quiescent state for most cells (Supplementary Fig. 3). The grafts had a complete endothelial cell layer, as evidenced by the uniform expression of Von Willebrand factor along both the pulmonary artery (Fig. 6g) and the entire length of the graft (Fig. 6h). The endothelial cells were also stained for E-selectin (CD62) to assess their activation state, with no evidence of E-selectin activation seen in any of the grafts or the adjacent pulmonary artery (Supplementary Fig. 4) even after using overnight incubation in antigen retrieval buffer. Further evidence of native-like tissue organization was observed when cells were imaged for calponin in both the circumferential and axial directions, showing elongated cells aligned in the circumferential direction (Supplementary Fig. 5). Evidence of a neo-adventitial layer including small vessels was evident on the ablumenal surface of the explanted grafts (Fig. 3d and Supplementary Fig 6).

## Discussion

Tissue engineering has the potential to overcome the limitations of existing treatments for congenital cardiovascular defects. An ideal treatment option would be durable, not prone to calcification, and possess the potential to somatic growth. To this end, two groups have previously demonstrated growth potential of synthetic biodegradable grafts seeded with autologous cells^13^,^22^,^23^,^24^. The studies by Shin'oka *et al.* led to a clinical trial in which 24 patients were enrolled^12^. While they have shown promising results, their approach relies on isolating and seeding the conduits with autologous cells before implantation. If growth and remodelling could be demonstrated with an ‘off-the-shelf' (acellular) conduit, this would simplify the procedure and the GMP-regulated manufacturing processes to prepare the grafts for clinical use.

Herein, we report an ‘off-the-shelf' pulmonary artery replacement capable of growing and remodelling. This study builds on our prior research, which used decellularized engineered tissue tubes as femoral artery grafts and tubular aortic heart valves in an adult sheep model. Both studies demonstrated excellent graft remodelling and function out to 24 weeks^27^,^28^ consistent with the pioneering studies in adult animals by Niklason and co-workers^29^. Based on the extensive recellularization and mechanical durability of the matrices found in our adult sheep studies, we undertook this study to investigate the growth potential of the matrix by implanting anatomically-matched 16 mm diameter grafts as a pulmonary artery replacement in lambs. The completely biological and circumferential alignment properties of these grafts are unique in comparison to grafts used in the aforementioned studies by Shin'oka co-workers, Hoerstrup co-workers and Niklasson co-workers among which only those of Niklasson *et al.* are ‘off-the-shelf', although not tested for growth potential. The 16 mm diameter grafts were evaluated for suture retention strength, which was comparable to the native artery and similar to that reported by Dahl *et al.*^29^. While, we did not measure burst pressure of our 16 mm grafts, their UTS was comparable to previously reported values for our 4 mm grafts fabricated using the same methods and with burst pressures exceeding 4,000 mm Hg (ref. 27). The animals were evaluated from average ages of 8 weeks to 50 weeks, with maximum graft implant duration of 44 weeks.

Over the course of the study, all animals were asymptomatic and showed healthy weight gain. During the study duration, the sheep increased in weight from 14 kg (8 week) to 67 kg (50 week), which was comparable to reported weight gain by non-surgical sheep^30^. To reduce the risk of clotting as a potential failure mode in our assessment of somatic growth potential of our tissue-engineered matrix, subdermal heparin was utilized for the duration of this study, based on our prior aortic valve implant experience^28^. No complications, bruising or bleeding were seen in any animal with anticoagulant therapy for the duration of study. Normal right heart function was observed with ultrasound 8 weeks post-surgery and at animal ages of 30 and 50 weeks.

Since we used biodegradable sutures that have a 4-week half-life, rapid host cell invasion and subsequent matrix deposition were necessary to fuse the anastomoses. Since we saw a uniform flow tract in all animals at all time points, it was apparent that the matrix fused with the pulmonary artery at the anastomoses before suture degradation. Although the exact timeline of cell invasion and fusion is not known for this study, we have previously shown that grafts harvested after 8 weeks in the femoral artery position had complete endothelial coverage near the anastomoses and invasion of α-SMA positive cells from the surrounding tissue^27^. Hence, it was not surprising in this study that sufficient extracellular matrix was deposited to ensure fusion at the anastomoses before the critical suture degradation point.

While the grafts were implanted as straight tubes, curvature around the aorta (as seen in the native pulmonary artery) was observed in the first ultrasound following implantation (8 weeks). Initially, this would most likely be due to physical forces on the graft; however, the cells invading the matrix remodelled the graft over time, while maintaining this physiological geometry.

Importantly, graft diameter and length increased over the duration of implantation, with diameter increased to the same degree as seen in the adjacent pulmonary artery. The uniform growth of the graft in all three animals contributed to the laminar flow profiles and normal pressure gradient across the grafts based on ultrasound examination. For comparison, in a growing lamb study using synthetic polytetrafluoroethylene (PTFE) conduits, a 20 mm Hg increase in pressure gradient developed within 1 year of implantation^31^. At the 50-week ultrasounds, it was challenging to visually discern the actual anastomotic locations as there was no narrowing, dilation or other markings to differentiate the graft and pulmonary artery, which had indistinguishable diameters. Evaluation of the explanted grafts confirmed uniform growth with diameters matching the adjacent artery and no difference in axial mechanical properties between the native artery above the graft, the anastomotic region, and the graft.

The explant graft volume increased by 216% over the course of implant, which was comparable to 244% volumetric increase of the pulmonary artery measured by Gottlieb *et al.* with magnetic resonance imaging^32^. For animal weight gains from 15 kg to 65 kg, they also reported cross-sectional area of the pulmonary artery increased from ∼300 mm^2^ to ∼600 mm^2^, or a ∼42% increase in diameter, which is also comparable to the 56% increase in diameter observed for our graft.

The strongest evidence of growth was seen when evaluating the mechanical property measurements and total collagen content together. No substantial change in mechanical properties between pre-implant and post-implant graft was seen even though the graft volume increased by 216%. This was most likely due to 465% more collagen measured in the explanted grafts, which appeared highly organized. Additionally, the new collagen was apparently deposited with the same circumferential alignment as the pre-implant graft, since strong mechanical anisotropy existed in the explanted tissue. In comparison, autologous cell-seeded polymeric grafts implanted by Hoerstrup *et al.*^23^ showed significantly higher stiffness of explanted grafts compared with pulmonary artery; however, no anisotropic properties were reported^23^. Although the collagen concentration in the explanted grafts (325–398 ug collagen mg^−1^ dry weight assuming a conversion factor from wet weight to dry weight of 0.20) was higher than in the explanted and control pulmonary arteries, it was in the range reported by Dahl *et al.* (420–570 ug mg^−1^) for their implanted and explanted engineered arteries^29^. This higher collagen content could explain the higher tensile modulus relative to the adjacent pulmonary artery. It is unknown whether it resulted in a lower compliance, as compliance was not measured in this study, and we relied on the tangent modulus for a direct comparison of intrinsic tissue stiffness (that is, at strains beyond which structural changes likely occur, albeit beyond physiological strains, independent of pre-load artifacts). Compliance mismatch can lead to turbulent flow through vascular grafts and intimal hyperplasia^33^,^34^; however, these were not observed, indicating any compliance mismatch was inconsequential.

Histologically, the graft exhibited substantial host cell invasion and deposition of matrix proteins comparable to the pulmonary artery. Although the pulmonary artery had ∼42% more cells, histological comparisons showed that the explanted grafts also contained mature, circumferentially-aligned smooth muscle cells and a complete endothelium with basement membrane. Further, the endothelial cells appeared to be in a non-activated phenotype (absence of E-selectin binding). While sustained heparin was used to ensure clotting would not occur, these results suggest it could have been terminated at some point before the explantation without risk of clotting due to the development of a non-activated endothelium. How this time-point could best be ascertained in the clinical setting is an interesting question. In a previous study of 4 mm diameter grafts implanted interpositionally into the sheep femoral artery, we removed all anticoagulants after 8 weeks of implantation and no evidence of thrombosis was observed at the 9-week explant^35^.

Histology of the explants also revealed an absence of two critical failure mechanisms, calcification (negligible von Kossa staining) and an immune response (negligible CD45 staining). Calcification of animal-derived valves and vessels treated with glutaraldehyde to mitigate an immunological response is a common failure mode, being particularly problematic in pediatric patients^36^. Since our ovine matrix was produced by a donor ovine cell entrapped in a bovine fibrin gel and cultured in FBS, the apparent absence of an immune response indicates the antigenic load following the decellularization plus any remnant fibrin or other bovine proteins was insufficient to elicit an immune response. Clinical use will require use of human dermal fibroblasts to minimize risk of a xenogeneic response to the cell-produced matrix; we have shown the ability to create human matrix tubes with properties comparable to ovine matrix tubes of the same diameter with this fabrication process^28^.

In our explanted grafts, elastin content was at 49% compared with pulmonary artery, with mature elastic fibers visible with Verhoeff's stain. In a previous growing lamb model, Hoerstrup *et al.*^23^ did not measure elastin and detected none via histology. Brennan *et al.*^22^ showed 47.2% more elastin deposition when synthetic polymer scaffold was pre-seeded with autologous cells compared with a cell-free scaffold. In comparison to pulmonary artery, Brennan *et al.*^22^ reported elastin content at 51% in their pre-seeded grafts after 26 weeks of implantation. Taking mature elastin as a marker for positive growth and remodelling, this acellular cell-produced matrix tube can thus remodel similar to pre-cellularized polymer grafts reported in previous studies. It is difficult to ascertain the contribution of the deposited elastin to the measured tensile mechanical properties because of the lack of specificity of elastase in treating tissue to selectively remove elastin (unpublished data). Notwithstanding, mechanical testing of elastase-treated arteries from a mouse model of Marfan syndrome in conjunction with a multi-fiber hyperelastic constitutive model was used to elucidate the role of elastin in load-bearing and undulation of collagen fibers^37^. Studies in newborn knockout mice lacking the elastin protein (Eln−/−) have shown dramatically increased arterial wall stiffness, and elastin haploinsufficient mice (Eln+/−) that produce only half of the elastin found in normal mice exhibit near-normal arterial wall stiffness at birth but postnatal remodelling and stiffening into adulthood^38^. Thus, it is not surprising that our explanted grafts exhibited tensile mechanical properties differing somewhat from the pulmonary artery.

Overall, this is the first report of an ‘off-the-shelf'' tissue-engineered vascular graft implanted in a growing lamb model that exhibited somatic growth and normal physiological function for nearly 1 year. All three implanted grafts were extensively recellularized (including complete endothelialization), with organized collagen and elastin deposition, and no evidence of calcification or aneurysm. They may thus serve as permanent conduits for pediatric cardiac vessel repair, reconstruction, and replacement.

## Methods

### Engineered tissue tubes

Ovine dermal fibroblast-seeded fibrin gels were formed by adding thrombin (Sigma) and calcium chloride in 20 mM HEPES-buffered saline to a suspension of cells (ovine dermal fibroblast from Coriell) and bovine fibrinogen (Sigma). The final component concentrations of the suspension were as follows: 4 mg ml^−1^ fibrinogen, 0.38 U ml^−1^ thrombin, 5.0 mM Ca^++^ and 1 million cells ml^−1^. The suspensions were mixed and injected into a tubular glass mold. The tubular grafts were cultured statically for 2-weeks and then transferred to custom pulsed-flow-stretch bioreactors for an additional 5-week maturation period using cyclic stretching with the strain amplitude increased from 3 to 7% at rate of 1% per week^39^.

Following bioreactor conditioning, the tubes were decellularized. First the tubes were placed on an orbital shaker at room temperature for 6 h with 1% sodium dodecyl sulfate (SDS, Sigma) followed by 1% Triton X-100 (Sigma) for 30 min, extensively washed with PBS for 2 weeks at 4 °C, and then incubated at 37 °C in 2 U ml^−1^ deoxyribonuclease (Worthington Biochemical, DR1) in DMEM supplemented with 10% FBS overnight. Grafts were sterilely stored at 4 °C until use in phosphate buffer solution.

### Graft implant in growing lamb model

Tissue-engineered ovine grafts were implanted as pulmonary artery replacements in *n*=3 Dorset lambs (average weight: 15.3 kg; average age at implant: 8.4 weeks; two male, one female). All protocols were approved by the Institutional Animal Care and Use Committee of the University of Minnesota and conform to NIH guidelines on Care and Use of Laboratory Animals. The surgeries were performed by the University of Minnesota's Experimental Surgical Services. For all animals, anesthesia was induced by administering 10 mg kg^−1^ Ketamine (intramuscular) and 2–6 mg kg^−1^ propofol (intravenously). Animals were then incubated and maintained on isoflurane at 1–3% for the duration of surgery and monitored for heart rate, mean blood pressure, fixed pupil location, corneal reflex absence and oxygen saturation to ensure proper anesthesia. The heart was exposed by a left lateral thoracotomy with dissection through the intercostal space. The animal was heparinized (250 IU kg^−1^, intravenously) and placed on cardiopulmonary bypass. The grafts were implanted interpositionally using continuous 5-0 Maxon CV (Covidien) degradable sutures after excising a similar length of the native main pulmonary artery. The native pulmonary valve was left intact. In addition, prolene sutures were used to attach two silver clips on the native pulmonary artery near the anastomoses to serve as markers (Fig. 1f).

Post-surgery, animals received subcutaneous 750 IU heparin BID for the duration of the study. For pain, animal received ketoprofen 1–2 mg kg^−1^ (intramuscular) every 12–24 h as directed by the post-operative veterinarian. Additionally slow release buphenorphine 0.27 mg kg^−1^ (SQ) was given before induction of anesthesia. Animals for the study were numbered as PAC1, PAC2 and PAC3. The first ultrasound was performed 8 weeks following implantation. The second and third ultrasounds were done when the animals were 30 weeks old and before euthanasia at 50 weeks of age, respectively. Conduit dimensions, pressure drop, flow velocity and flow profile (laminar or turbulent characteristics) were measured from the ultrasound. Diameter and flow velocity were measured mid-graft, and pressure drop was determined from velocity measurements mid-graft and just beyond the distal anastomosis. Animals were heparinized (300 IU kg^−1^, intravenously) and then euthanized with beuthanasia given intravenously at 87–90 mg kg^−1^. Explanted grafts were photographed, cleaned of loose connective tissue, and then dissected into strips for histological, biochemical and mechanical characterization. *N*=3 non-surgical aged-match (50 week old) sheep hearts were also collected and right ventricle wall thickness and pulmonary artery diameter were measured. Samples from these control arteries were also evaluated for tensile mechanical properties, biochemical analysis and immunohistochemistry.

### Mechanical testing

Tissue strips were cut from the engineered tissue tube before implant (*n*=5) and following explant (*n*=3) with dimensions of ∼2 mm × 10 mm in both the circumferential and axial directions. In addition, non-surgical control samples were collected from the age-matched control sheep (*n*=3) and the explanted pulmonary artery (*n*=3). Following explant, additional axial strips were cut that consisted of half native pulmonary artery and half engineered tissue from the anastomotic regions. All samples were measured for dimensions and then tested for tensile mechanical properties using an Instron mechanical testing system and compression grips. The sample was stretched at constant rate of 10 mm min^−1^ (strain rate of ∼5% per second). The tangent modulus (E) was defined as the slope of the linear region of the stress–strain curve before failure. The peak stress was defined as UTS. Maximum tension was defined as the UTS multiplied by the average thickness and used in case all layers of a sample do not bear equal amounts of load^40^. Mechanical anisotropy was defined as the ratio of the modulus of tissue samples cut in the circumferential direction to the modulus of samples cut from the tissue in the axial direction.

Suture tension properties were evaluated in accordance with ISO 7198. Briefly, a 7-0 prolene suture (Ethicon Cat#8648G) was passed through a circumferential tissue strip (∼10 mm × 10 mm) 2 mm from the free edge. The suture was tied into a loop and pulled at 50 mm min^−1^ axially at constant rate until failure using an Instron mechanical testing system. The maximum force before rupture was defined as suture retention force. A thinner 7-0 suture was used, instead of 5-0 suture as used for surgery, to give a conservative estimate of retention force.

### Tissue composition and DNA analysis

The collagen mass content was quantified using a hydroxyproline assay previously described^41^ assuming 7.46 mg of collagen per 1 mg of hydroxyproline. Insoluble elastin was measured by dissolving tissue strip samples in NaOH and using a modified ninhydrin assay to measure elastin^42^. The total protein content was measured using the ninhydrin assay^43^. The tissue volume was calculated using the measured length, width and thickness of the samples (reported in units of ml) was then used to calculate the sample mass concentration (mg protein ml^−1^ tissue) of collagen, total protein and elastin. For total mass content of the pre-implant grafts (*n*=5) and explanted grafts (*n*=3) and the control and explanted pulmonary artery segments (*n*=3), length, width and thickness of the entire graft or arterial segment were used to measure total volume, which was then multiplied by the respective sample mass concentration (collagen, total protein or elastin). The DNA content was quantified with a modified Hoechst assay for total DNA^44^.

### Histology and immunostaining

Each explanted graft was histologically and immunologically stained; multiple strips were cut to cover all regions of interest. Circumferential and axial tissue strips of pre-implant and explanted grafts were fixed in 4% paraformaldehyde, embedded in OCT (Tissue-Tek), and frozen in liquid N_2_. Cross sections of 9-μm thickness were stained with Lillie's trichrome, Verhoeff-Van Gieson elastin stain, and picrosirius red stain. Histological sections were also immunostained for α-SMA (Sigma, A5228, clone#ACTA2, monoclonal Mouse, 1:200 dilution), Calponin (Abcam ab46794, clone# EP798Y, monoclonal Rabbit, 1:100 dilution), vimentin (Abcam, ab80667, clone#V9, monoclonal Mouse, 1:1000 dilution), Smoothelin (Calbiochem 400080Clone#R4A, monoclonal Mouse, 1:100 dilution), Von Willebrand Factor (Abcam ab6994, polyclonal Rabbit, 1:200 dilution), E-selectin (CD62E, Abcam, ab122964, polyclonal Rabbit, 1:200 dilution), CD45 (US Biological C2399-07B, clone#10B1601, monoclonal Mouse, 1:200 dilution), Ki67 (Abcam ab15580, polyclonal Rabbit, 1:400 dilution), elastin (Abcam ab21599, clone#BA4, monoclonal Mouse, 1:1000 dilution) and collagen IV (Abcam, ab6586, polyclonal Rabbit, 1:200 dilution). All samples were blocked with 5% normal donkey serum, incubated in primary antibody at 2.5–5 ug ml^−1^ and stained with a Cy5-conjugated, species-matched secondary antibody (Jackson Immunoresearch). Nuclei were counterstained with Hoechst 33342 (Invitrogen H3570). Positive controls For E-Selectin staining were generated by incubating a fresh section of sheep femoral artery (1 cm segment) and a monolayer of cultured endothelial cells in a 48-well plate overnight with tumour-necrosis factor-α (R&D Systems 210-TA) at a concentration of 50 ng ml^−1^ in EBM-2 medium (Lonza CC-3156).

### Statistics

Average value are plotted with ‘*n*' value shown in figure legend. The s.d. is reported as error bars for all averaged values. Statistical significance for differences between two groups was determined using Student's *t*-test when comparing two groups and analysis of variance with Tukey *post-hoc* analysis for more than two groups. Paired symbols are used in figures to represent statistical difference between two groups. Any reference to a difference in the Results and Discussion sections implies statistical significance at the level *P*<0.05.

### Data availability

All relevant data supporting the findings of this study are either included within the article and its Supplementary Information files or available upon request from the corresponding author.

## Additional information

**How to cite this article:** Syedain, Z. *et al.* Tissue engineering of acellular vascular grafts capable of somatic growth in young lambs. *Nat. Commun.* **7,** 12951 doi: 10.1038/ncomms12951 (2016).

## Supplementary Material

Supplementary Information Supplementary Figures 1-6.

## Figures and Tables

**Table 1:** Measured dimensions of grafts at implant and explant.

Graft	Implant diameter (mm)	Explant diameter (mm)	Implant length (mm)	Explant length (mm)
PAC1	16	24.7	19	41
PAC2	16	24.1	16	39
PAC3	16	24.0	16	37

## References

[b1] DiBardino D. J. & Jacobs J. P. Current readings: long-term management of patients undergoing successful pediatric cardiac surgery. Semin. Thorac. Cardiovasc. Surg. 26, 132–144 (2014). 25441004 10.1053/j.semtcvs.2014.08.002

[b2] Downing T. E. & Kim Y. Y. Tetralogy of fallot: general principles of management. Cardiol. Clin. 33, 531–541 (2015). 26471818 10.1016/j.ccl.2015.07.002

[b3] Yuan S. M., Mishaly D., Shinfeld A. & Raanani E. Right ventricular outflow tract reconstruction: valved conduit of choice and clinical outcomes. J. Cardiovasc. Med. (Hagerstown) 9, 327–337 (2008). 18334887 10.2459/JCM.0b013e32821626ce

[b4] Jacobs J. P. *et al.* Reoperations for pediatric and congenital heart disease: an analysis of the Society of Thoracic Surgeons (STS) congenital heart surgery database. Semin. Thorac. Cardiovasc. Surg. Pediatr. Card. Surg. Annu. 17, 2–8 (2014). 24725711 10.1053/j.pcsu.2014.01.006 PMC4276147

[b5] Weber B. *et al.* Off-the-shelf human decellularized tissue-engineered heart valves in a non-human primate model. Biomaterials 34, 7269–7280 (2013). 23810254 10.1016/j.biomaterials.2013.04.059

[b6] Mol A., Smits A. I., Bouten C. V. & Baaijens F. P. Tissue engineering of heart valves: advances and current challenges. Expert Rev. Med. Devices 6, 259–275 (2009). 19419284 10.1586/erd.09.12

[b7] Gottlieb D. *et al.* *In vivo* monitoring of function of autologous engineered pulmonary valve. J. Thorac. Cardiovasc. Surg. 139, 723–731 (2010). 20176213 10.1016/j.jtcvs.2009.11.006 PMC12998794

[b8] Funayama M. *et al.* *In situ* observation and enhancement of leaflet tissue formation in bioprosthetic ‘biovalve'. J. Artif. Organs 18, 40–47 (2015). 25370717 10.1007/s10047-014-0793-x

[b9] Reimer J. M., Syedain Z. H., Haynie B. H. & Tranquillo R. T. Pediatric tubular pulmonary heart valve from decellularized engineered tissue tubes. Biomaterials 62, 88–94 (2015). 26036175 10.1016/j.biomaterials.2015.05.009 PMC4490908

[b10] Robinson P. S. & Tranquillo R. T. Planar biaxial behavior of fibrin-based tissue-engineered heart valve leaflets. Tissue Eng. Part A 15, 2763–2772 (2009). 19368523 10.1089/ten.tea.2008.0426 PMC2792051

[b11] Shin'oka T., Imai Y. & Ikada Y. Transplantation of a tissue-engineered pulmonary artery. N. Engl. J. Med. 344, 532–533 (2001). 11221621 10.1056/NEJM200102153440717

[b12] Shin'oka T. *et al.* Midterm clinical result of tissue-engineered vascular autografts seeded with autologous bone marrow cells. J. Thorac. Cardiovasc. Surg. 129, 1330–1338 (2005). 15942574 10.1016/j.jtcvs.2004.12.047

[b13] Hibino N. *et al.* Late-term results of tissue-engineered vascular grafts in humans. J. Thorac. Cardiovasc. Surg. 139, 431–436 (2010). 20106404 10.1016/j.jtcvs.2009.09.057

[b14] Matsumura G. & Shinoka T. First report of histological evaluation of human tissue-engineered vasculature. J. Biotechnol. Biomater. 5:3 (2015).

[b15] Hibino N. *et al.* The innate immune system contributes to tissue-engineered vascular graft performance. FASEB J. 29, 2431–2438 (2015). 25713026 10.1096/fj.14-268334 PMC4447224

[b16] Tara S. *et al.* Evaluation of remodeling process in small-diameter cell-free tissue-engineered arterial graft. J. Vasc. Surg. 62, 734–743 (2015). 24745941 10.1016/j.jvs.2014.03.011

[b17] James I. A. *et al.* Hemodynamic characterization of a mouse model for investigating the cellular and molecular mechanisms of neotissue formation in tissue-engineered heart valves. Tissue Eng. Part C Methods 21, 987–994 (2015). 25915105 10.1089/ten.tec.2015.0011 PMC4553370

[b18] Roh J. *et al.* Tissue-engineered vascular grafts transform into mature blood vessels via an inflammation-mediated process of vascular remodeling. Proc. Natl Acad. Sci. USA 107, 4669–4674 (2010). 20207947 10.1073/pnas.0911465107 PMC2842056

[b19] Harrington J. K. *et al.* Determining the fate of seeded cells in venous tissue-engineered vascular grafts using serial MRI. FASEB J. 25, 4150–4161 (2011). 21846838 10.1096/fj.11-185140 PMC3236630

[b20] Hibino N. *et al.* Tissue-engineered vascular grafts form neovessels that arise from regeneration of the adjacent blood vessel. FASEB J. 25, 2731–2739 (2011). 21566209 10.1096/fj.11-182246 PMC3136337

[b21] Hibino N. *et al.* A critical role for macrophages in neovessel formation and the development of stenosis in tissue-engineered vascular grafts. FASEB J. 25, 4253–4263 (2011). 21865316 10.1096/fj.11-186585 PMC3236622

[b22] Brennan M. P. *et al.* Tissue-engineered vascular grafts demonstrate evidence of growth and development when implanted in a juvenile animal model. Ann. Surg. 248, 370–377 (2008). 18791357 10.1097/SLA.0b013e318184dcbd PMC2726802

[b23] Hoerstrup S. P. *et al.* Functional growth in tissue-engineered living, vascular grafts: follow-up at 100 weeks in a large animal model. Circulation 114, I159–I166 (2006). 16820566 10.1161/CIRCULATIONAHA.105.001172

[b24] Kelm J. M. *et al.* Functionality, growth and accelerated aging of tissue engineered living autologous vascular grafts. Biomaterials 33, 8277–8285 (2012). 22906604 10.1016/j.biomaterials.2012.07.049

[b25] Rensen S. S., Doevendans P. A. & van Eys G. J. Regulation and characteristics of vascular smooth muscle cell phenotypic diversity. Neth. Heart J. 15, 100–108 (2007). 17612668 10.1007/BF03085963 PMC1847757

[b26] Christen T. *et al.* Mechanisms of neointima formation and remodeling in the porcine coronary artery. Circulation 103, 882–888 (2001). 11171799 10.1161/01.cir.103.6.882

[b27] Syedain Z. H., Meier L. A., Lahti M. T., Johnson S. L. & Tranquillo R. T. Implantation of completely biological engineered grafts following decellularization into the sheep femoral artery. Tissue Eng. Part A 20, 1726–1734 (2014). 24417686 10.1089/ten.tea.2013.0550 PMC4029045

[b28] Syedain Z. *et al.* 6-month aortic valve implantation of an off-the-shelf tissue-engineered valve in sheep. Biomaterials 73, 175–184 (2015). 26409002 10.1016/j.biomaterials.2015.09.016 PMC5520964

[b29] Dahl S. L. *et al.* Readily available tissue-engineered vascular grafts. Sci. Transl. Med. 3, 68ra69 (2011). 10.1126/scitranslmed.3001426 21289273

[b30] Fata B., Gottlieb D., Mayer J. E. & Sacks M. S. Estimated in vivo postnatal surface growth patterns of the ovine main pulmonary artery and ascending aorta. J. Biomech. Eng. 135, 71010–71012 (2013). 23757175 10.1115/1.4024619 PMC3705821

[b31] Molina J. E. *et al.* Composite and plain tubular synthetic graft conduits in right ventricle-pulmonary artery position: fate in growing lambs. J. Thorac. Cardiovasc. Surg. 110, 427–443 (1995). 7637361 10.1016/S0022-5223(95)70239-3

[b32] Gottlieb D. *et al.* Pulmonary artery conduit in vivo dimensional requirements in a growing ovine model: comparisons with the ascending aorta. J. Heart Valve Dis. 22, 195–203 (2013). 23798208

[b33] Miyawaki F., How T. V. & Annis D. Effect of compliance mismatch on flow disturbances in a model of an arterial graft replacement. Med. Biol. Eng. Comput. 28, 457–464 (1990). 2277546 10.1007/BF02441969

[b34] Abbott W. M., Megerman J., Hasson J. E., L'Italien G. & Warnock D. F. Effect of compliance mismatch on vascular graft patency. J. Vasc. Surg. 5, 376–382 (1987). 3102762

[b35] Meier L. A. *et al.* Blood outgrowth endothelial cells alter remodeling of completely biological engineered grafts implanted into the sheep femoral artery. J. Cardiovasc. Transl. Res. 7, 242–249 (2014). 24429838 10.1007/s12265-013-9539-z PMC4213739

[b36] Schoen F. J. & Levy R. J. Calcification of tissue heart valve substitutes: progress toward understanding and prevention. Ann. Thorac. Surg. 79, 1072–1080 (2005). 15734452 10.1016/j.athoracsur.2004.06.033

[b37] Ferruzzi J., Collins M. J., Yeh A. T. & Humphrey J. D. Mechanical assessment of elastin integrity in fibrillin-1-deficient carotid arteries: implications for Marfan syndrome. Cardiovasc. Res. 92, 287–295 (2011). 21730037 10.1093/cvr195 PMC3193833

[b38] Wagenseil J. E. *et al.* Reduced vessel elasticity alters cardiovascular structure and function in newborn mice. Circ. Res. 104, 1217–1224 (2009). 19372465 10.1161/CIRCRESAHA.108.192054 PMC2800958

[b39] Syedain Z. H., Meier L. A., Bjork J. W., Lee A. & Tranquillo R. T. Implantable arterial grafts from human fibroblasts and fibrin using a multi-graft pulsed flow-stretch bioreactor with noninvasive strength monitoring. Biomaterials 32, 714–722 (2011). 20934214 10.1016/j.biomaterials.2010.09.019 PMC3042747

[b40] Billiar K. & Sacks M. Biaxial mechanical properties of the natural and glutaraldehyde treated aortic valve cusp - part I: experimental results. J. Biomech. Eng. 122, 23–30 (2000). 10790826 10.1115/1.429624

[b41] Stegemann H. & Stalder K. Determination of hydroxyproline. Clin. Chim. Acta 18, 267–273 (1967). 4864804 10.1016/0009-8981(67)90167-2

[b42] Starcher B. C. & Galione M. J. Purification and comparison of elastins from different animal species. Anal. Biochem. 74, 441–447 (1976). 822746 10.1016/0003-2697(76)90224-4

[b43] Robinson P. S., Johnson S. L., Evans M. C., Barocas V. H. & Tranquillo R. T. Functional tissue-engineered valves from cell-remodeled fibrin with commissural alignment of cell-produced collagen. Tissue Eng. Part A 14, 83–95 (2008). 18333807 10.1089/ten.a.2007.0148

[b44] Kim Y. J., Sah R. L., Doong J. Y. & Grodzinsky A. J. Fluorometric assay of DNA in cartilage explants using Hoechst 33258. Anal. Biochem. 174, 168–176 (1988). 2464289 10.1016/0003-2697(88)90532-5

