# Corrigendum: Tissue engineering of acellular vascular grafts capable of somatic growth in young lambs

**DOI:** 10.1038/ncomms14297

**Published:** 2017-01-17

**Authors:** Zeeshan Syedain, Jay Reimer, Matthew Lahti, James Berry, Sandra Johnson, Richard Bianco, Robert T. Tranquillo

Nature Communications 7: Article number: 12951; DOI: 10.1038/ncomms12951 (2016); Published 11 25 2015; Updated 12 17 2017

The authors inadvertently omitted Richard Bianco, who provided consultation for the experimental surgical services, from the author list. This has now been corrected in both the PDF and HTML versions of the Article.

