# Supplementary material for: Tissue engineering of acellular vascular grafts capable of somatic growth in young lambs
Source: Nat Commun. 2016 Sep 27;7:12951. doi: 10.1038/ncomms12951 (PMC5052664; doi:10.1038/ncomms12951)

Suppl. Figure 1: Ultrasound images of the graft for PAC1 (top panel), PAC2 (middle panel) and PAC3 (bottom panel) at 8 weeks post-implantation and at animal age of 30 and 50 weeks. White arrows mark the anastomotic positions. 'A' marks the lumen of the aorta.

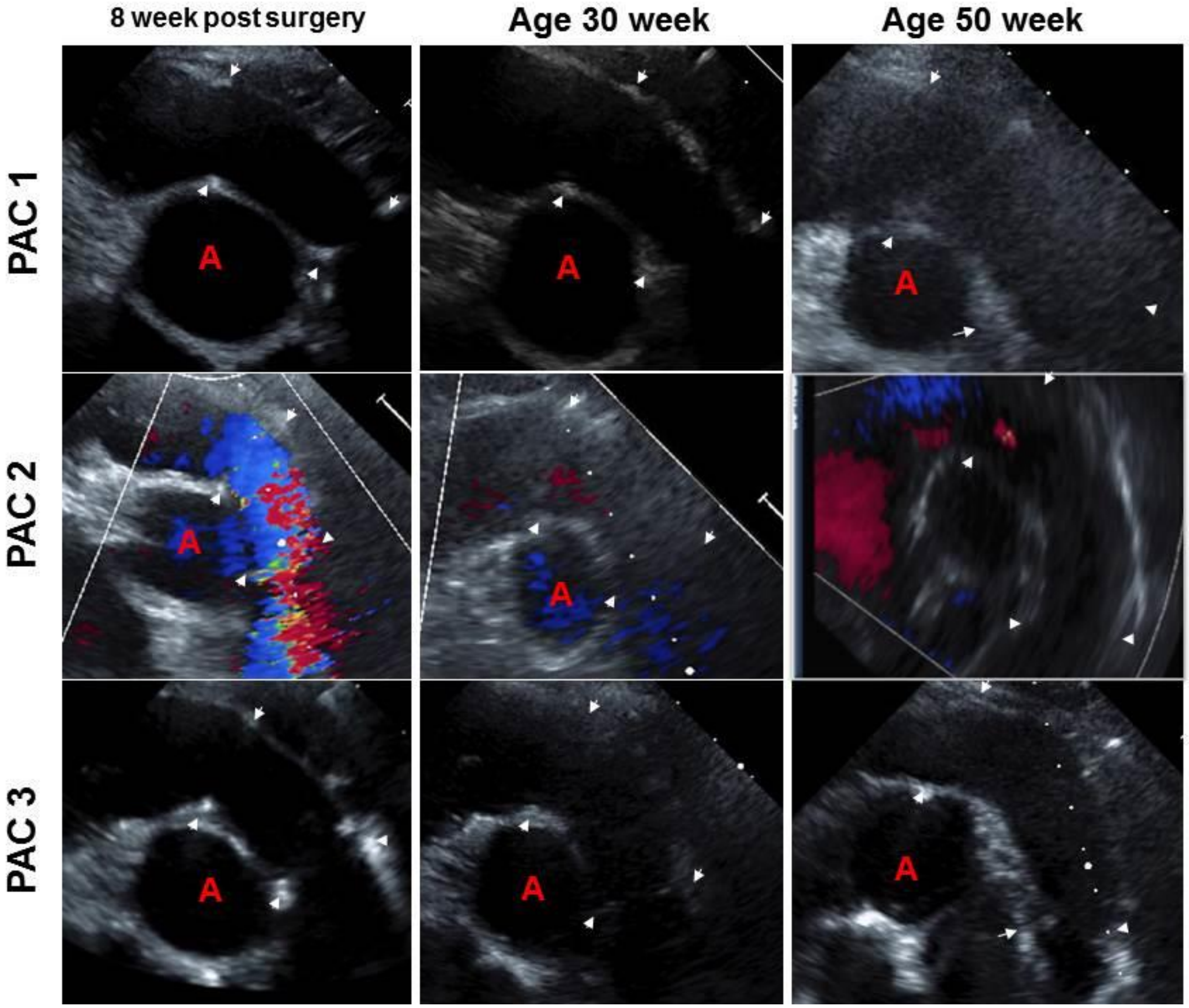

Suppl. Figure 2: Images of grafts at implant a. PAC1, b. PAC2, and c. PAC3, at explant d. PAC1, e. PAC2 and f. PAC3. Images of cross-section of right ventricle showing ventricle wall thickness for **g.** explanted heart and **h.** non-surgical control. RV: Right Ventricle, LV: Left Ventricle.

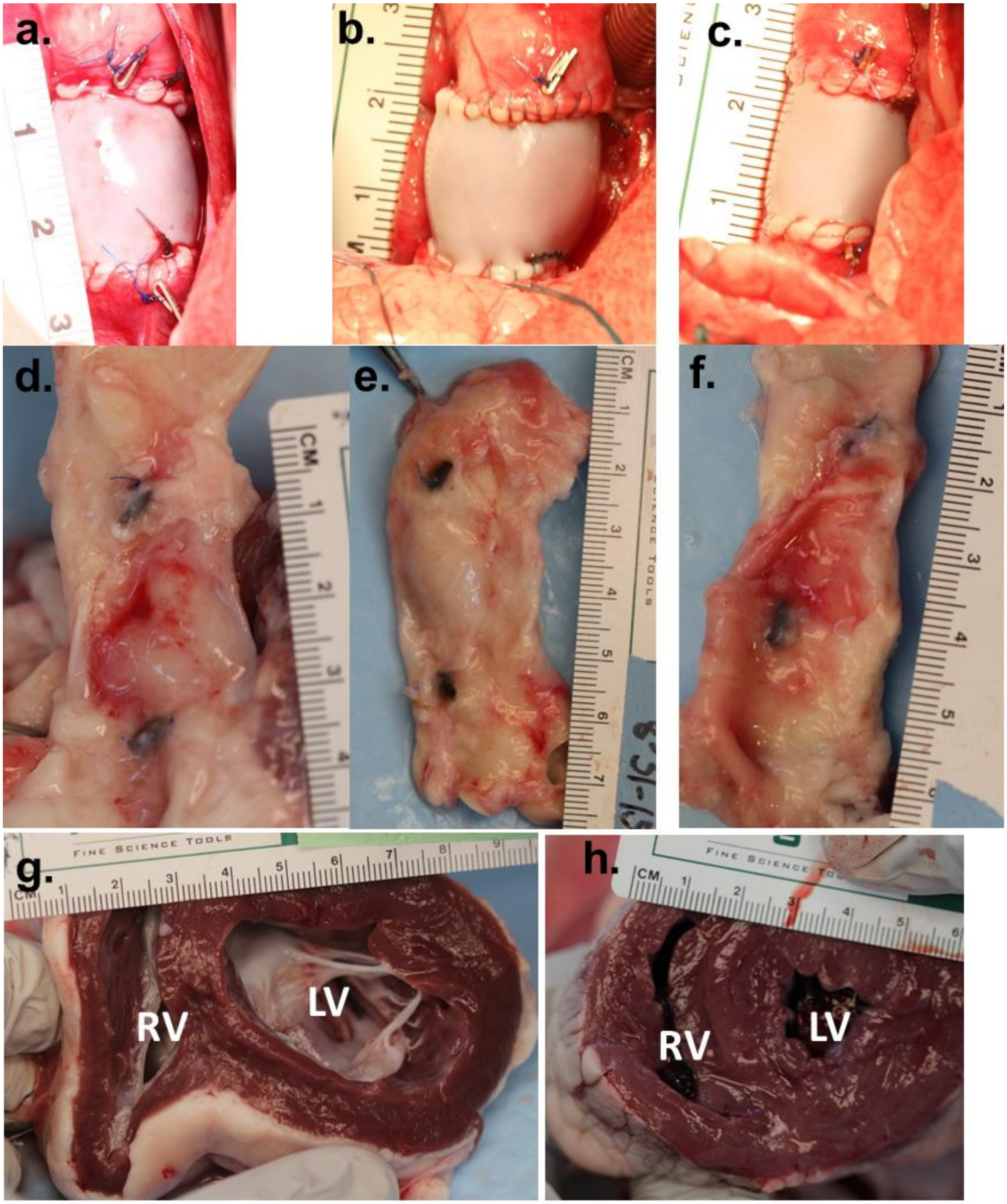

Suppl. Figure 3: Ki67 immunostaining for cell proliferation in the **a.** explanted pulmonary artery and **b.** the explanted graft. 200 μm scalebars shown.

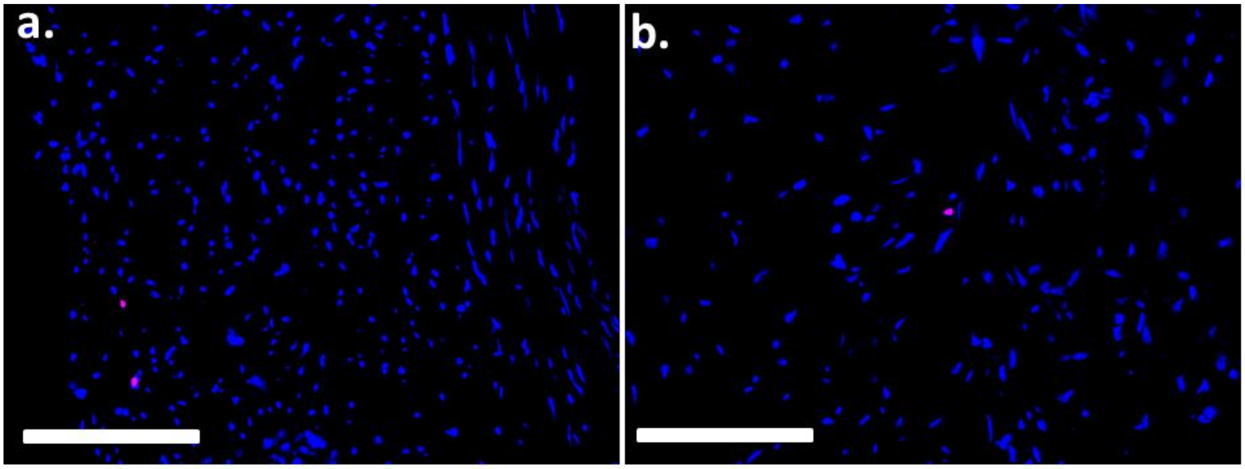

Suppl. Figure 4: E-selectin (CD62) immunostaining of the **a**. Untreated cultured endothelial cells, **b.** TNF-α treated cultured endothelial cells, **c.** TNF-α treated control artery (IgG control), **d.** TNF-α pretreated control artery, **e.** native pulmonary artery adjacent to the explanted graft, **f.** PAC1, **g.** PAC2, and **h.** PAC3. In (d), the E-selectin staining was co-localized with vWF staining (not shown) but the endothelium was incomplete due to partial detachment during the overnight TNF-α pretreatment. '*' marks the lumenal side. 50 μm scalebars shown.

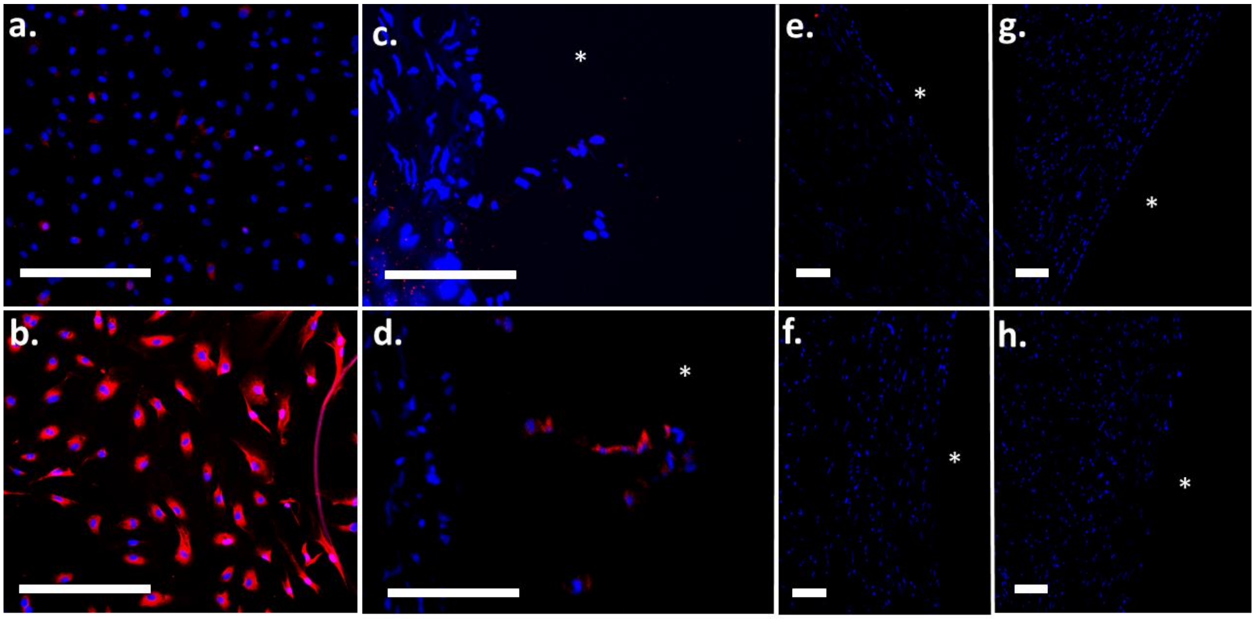

Suppl. Figure 5: Calponin immunostaining of the explanted graft in the **a.** circumferential and **b.** axial directions showing elongated cells in the circumferential direction. 200 μm scalebars shown. '*' marks the lumenal side.

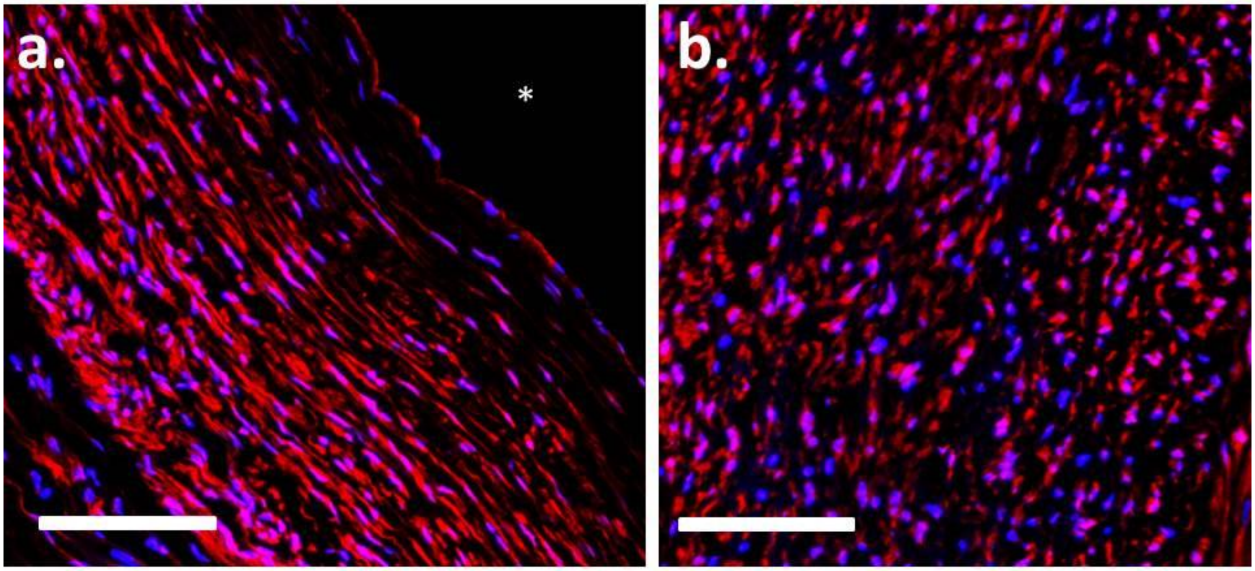

Suppl. Figure 6: Trichrome image comparison of **a.** control pulmonary artery, and **b.** explanted graft at 50 weeks, with the white dashed line demarcating the graft from the neo-adventitial layer spontaneously growing on the ablumenal surface **c.** vWF stain for endothelial cells showing small vessels in the neo-adventitial layer (marked with white arrow). (a-c) are axial sections. '*' marks the lumenal side. 200 μm scalebars shown.

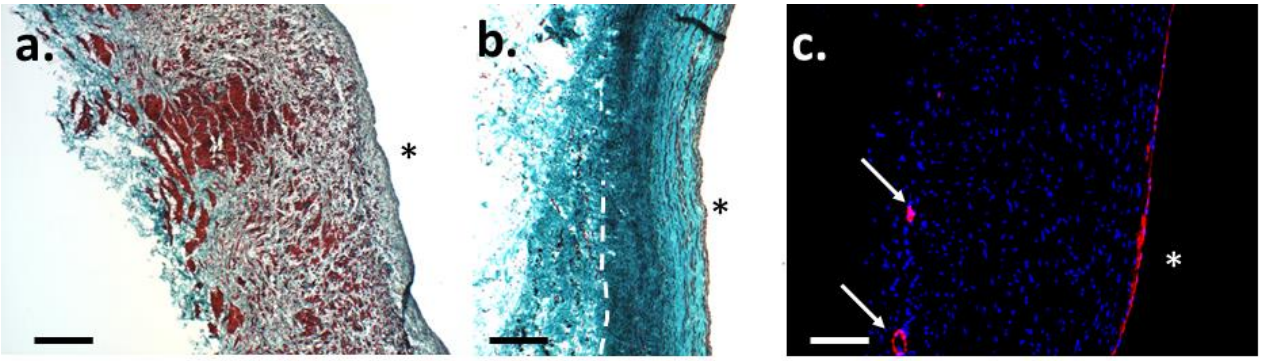

Supplement: Supplementary Information — Supplementary Figures 1-6. [file ncomms12951-s1.pdf]
